# Is the Development of Ascites in Alcoholic Liver Patients Influenced by Specific KIR/HLA Gene Profiles?

**DOI:** 10.3390/biomedicines11092405

**Published:** 2023-08-28

**Authors:** Isabel Legaz, Raquel Morales, José Miguel Bolarín, Aurelia Collados-Ros, José Antonio Pons, Manuel Muro

**Affiliations:** 1 Department of Legal and Forensic Medicine, Biomedical Research Institute of Murcia (IMIB), Regional Campus of International Excellence “Campus Mare Nostrum”, Faculty of Medicine, University of Murcia (UMU), 30100 Murcia, Spainbolarin5@hotmail.com (J.M.B.);; 2 Department of Hepatology, Liver Transplantation Unit Hospital Clinic Universitario, Virgen de la Arrixaca, IMIB-Arrixaca, 30120 Murcia, Spain; 3 Immunology Service, University Clinical Hospital “Virgen de la Arrixaca”—IMIB, 30120 Murcia, Spain

**Keywords:** alcoholic cirrhosis, ascites, human clinical toxicology, liver transplant, KIR/HLA-C genes

## Abstract

Decompensated cirrhosis is the most common cause of ascites due to hemodynamic and renal alteration by continuous fluid leakage from the hepatic sinusoids and splanchnic capillaries into the interstitial space. Then, fluid leakage exceeds lymphatic return, leading to progressive fluid accumulation directly into the peritoneal cavity. Alcohol consumption is one of the main risks of developing alcoholic cirrhosis (AC), but not all AC patients develop ascites. Avoiding the development of ascites is crucial, given that it deteriorates prognosis and increases the patient mortality patient. The innate immune system plays a crucial role in cirrhosis through natural killer cells, which are abundant in the liver. The aim of this study was to analyze the KIR/HLA-C genetic profile in AC patients with and without ascites to understand this pathology and find predictive clinical susceptibility biomarkers that can help to establish risks and prevent the development of ascites in AC patients. A total of 281 AC patients with and without ascites were analyzed and compared with 319 healthy controls. Genomic DNA was extracted from peripheral blood in all groups. A PCR-SSO assay was performed for KIR/HLA genotyping analysis. A total of 16 activating and inhibitor KIR genes and their corresponding known ligands, epitopes of HLA-C, and their genotypes were analyzed. According to our analysis, C1 epitopes were statistically significantly decreased in AC patients with and without ascites. When comparing AC patients with ascites and healthy controls, a significant decrease in C1 epitope frequency was also observed. A statistically significant decrease was also found when comparing the C1C2 genotype in AC patients without ascites with controls. In conclusion, the absence of KIR2DL2 and KIR3DL1 genes may be a predisposing factor for the development of ascites in AC patients. The KIR2DS2/KIR2DL2 may could be involved in grade I ascites development, and the presence of the C1+ epitope and the homozygous C2C2 genotype may be protective genetic factors against ascites development in AC patients.

## 1. Introduction

Ascites represents a critical event in the natural history of liver cirrhosis [1,2], deteriorating prognosis and increasing mortality rates [1,2]. Cirrhosis causes hemodynamic and renal alteration by continuous fluid leakage from the hepatic sinusoids and splanchnic capillaries into the interstitial space [3,4]. As cirrhosis progresses, fluid leakage exceeds lymphatic return, leading to progressive fluid accumulation directly into the peritoneal cavity, producing ascites in cirrhotic patients [5,6,7,8]. Other complications associated with portal hypertension include spontaneous bacterial peritonitis (SBP), hepatic encephalopathy (HE), hepatorenal syndrome, portopulmonary hypertension, or variceal bleeding [9,10,11]. Effective hypovolemia serves as the critical event in the pathophysiology of ascites, which traditionally relies on hemodynamic mechanisms [12]. Patients with decompensated cirrhosis in the final stages of their illness are generally indicated for liver transplantation (LT) [13]. 

Diverse studies have identified immune system deregulation and systemic inflammation as the primary processes in ascites [3,14]. Natural killer (NK) cells have been studied in lymphocyte-associated ascites [15,16]. NK lymphocytes often participate in the immune defense against malignant or virally infected cells, attacking such changed cells directly and contributing in the development of the adaptive response through cytokine production and interactions with other immune system cells [17,18]. In contrast, later stages of ovarian cancer are also marked by the presence of ascites [19,20]. Compared to ascites in advanced ovarian cancer and liver cirrhosis, some studies observed selectively concentrated CD4+CD25+ lymphocytes and NK-like T lymphocytes (CD3+CD56+) in the peritoneal cavity [11]. Ascites also considerably increase these cells in the peritoneal cavity relative to blood [19,20]. The effector activity of NK cells is regulated by the balance between activation and inhibition signals as a result of the expression of killer cell immunoglobulin-like receptor (KIR) molecules with different functional properties and human leukocyte antigen (HLA) class I molecules [21]. Recent studies on KIR-like receptors and HLA ligands suggest that they play an interesting role in liver immunopathology [22,23]. 

The aim of this study was to analyze the KIR/HLA-C genetic profile in AC patients with and without ascites to clarify the understanding of this pathology and find predictive clinical susceptibility biomarkers that can help to establish risks and prevent ascites development in AC patients.

## 2. Patients and Methods

### 2.1. Patient Enrollment

The medical records of 281 AC patients in the final stage of cirrhosis on the waiting list for a liver transplant (LT) at the University Clinic Hospital Virgen de la Arrixaca (Spain) were analyzed retrospectively. Sociodemographic data (age and sex), the presence or absence of ascites, and the KIR/HLA genetic profile were studied. All clinical parameters analyzed in this study were collected when our patients were first enrolled on the LT waiting list. The model for end-stage liver disease score in the AC patients on the waiting list for LT was 14.23 ±0.37 (mean ± SEM). The analyzed patients had a mean age of 53.02 ± 0.43 years (mean years ± SEM). The inclusion criteria were HIV-negative male AC patients without prior history of other organ transplants. A total of 319 male individuals matched in age were included as healthy controls for comparison with male AC patients. 

All patients provided informed consent for inclusion before participating in the study. The study was conducted following the Declaration of Helsinki and was approved by the Ethics Committee of HUVA (PI19/01194).

### 2.2. Diagnostic Criteria of Alcohol Cirrhosis 

Clinical, radiographic, and biochemical markers were used to diagnosis AC [24]. In the case of a negative self-report of alcoholic beverage consumption, the relatives’ opinion was considered. Cirrhosis typically has no symptoms in the early stages; thus, a routine scan, ultrasound, or clinical examination is used to make the diagnosis. In some cases, the disease went undetected until the second stage of decompensated cirrhosis, when symptoms such ascites, upper gastrointestinal bleeding, and encephalopathy became apparent. Cases of probable cirrhosis were confirmed using particular analysis and imaging approaches, as previously reported [25].

### 2.3. Ascites Diagnosis

**Figure 1:**
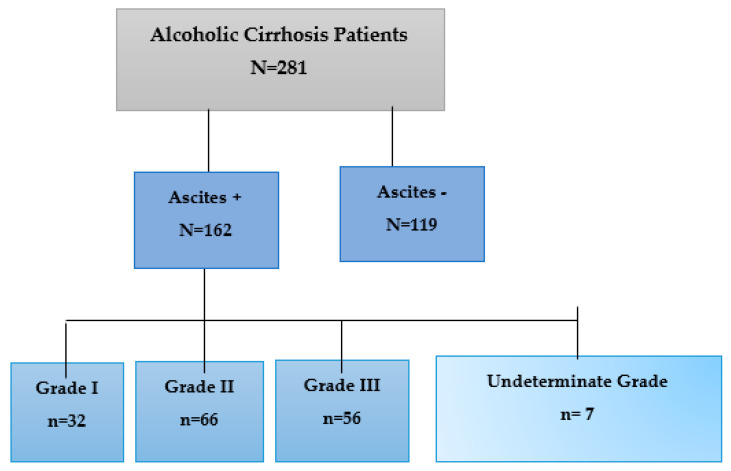
Schematic of the types of patients analyzed in this study.

Ascites was evaluated in 281 medical records of male AC patients when entering the waiting list for LT (Figure 1). Ascites was diagnosed by history; physical examination; and imaging tests, such as abdominal ultrasound [26], tomography, or magnetic resonance, establishing three grades ranging from low (grade I) to high involvement (grade II and III) [27,28]. In some cases, it was not possible to determine the degree of ascites (7/162).

### 2.4. Biochemical Parameters Analyzed in AC Patients with and without Ascites

A total of nine biochemical parameters were analyzed in this study based on previously published normalized values [29].

### 2.5. KIR and HLA Typing 

Genomic DNA from peripheral blood was extracted using a QIAamp DNA Blood Mini Kit (QIAGEN, Hilden, Germany), as recommended by the manufacturer and previously published [22]. KIR genotyping was performed in patients and controls by Luminex^®^ technology (Tepnel Lifecodes, Stanford, CT, USA) using sequence-specific oligonucleotides (PCR-SSO). This method identifies inhibitory (iKIR) KIR2DL1-3 and KIR3DL1-3, activating (aKIR) KIR2DS1-S5 and KIR3DS1 genes and KIR2DL4. The KIR2DL5A and KIR2DL5B genes could not be distinguished, while KIR2DP1 and KIR3DP1 pseudogenes were identified but not analyzed in this study. Since KIR2DL2 is generally closely associated with KIR2DS2 and individual effects could not be distinguished in our population, both genes were studied. HLA class I genotyping was also performed using PCR-SSO by Luminex^®^ (Tepnel Lifecodes, Stanford, CT, USA), with a resolution level that identified the dimorphism at position 80 with HLA-C alleles assigned to C1 or C2 genotypes, as previously published [30,31]. Their study established three genotypes: homozygous C1C1 and C2C2 and heterozygous C1C2.

### 2.6. Statistical Analysis

Demographic and outcome data were entered into a database (Microsoft Access 2.0; Microsoft Corporation, Seattle, WA, USA) and analyzed with SPSS v27.0 (SPSS software Inc., Chicago, IL, USA). All data were reported as the mean, standard deviation, or percentage. Pearson’s chi-square and two-tailed Fisher’s exact tests were employed to compare classified variables between groups, while a two-sided Student’s *t*-test and a non-parametric Mann–Whitney test were used to compare mean values. The statistical significance threshold was set at *p* < 0.05. The odds ratio (OR) and 95% confidence interval (CI) were determined to evaluate relative risk. 

## 3. Results

### 3.1. Sociodemographic and Clinical Characteristics

The sociodemographic and main clinical characteristics of the total cohort (n = 281) of male AC patients are shown in Table 1. Pediatrics and patients in whom specific tests could not be conducted were excluded from this study. The mean age immediately prior to transplant was similar (53.02 ± 0.43 years (mean years ± SEM)) in all analyzed patients. The inclusion criteria were HIV-negative male patients with alcohol liver cirrhosis and without a history of organ transplants.

### 3.2. Biochemical Characteristics of the AC Patient with and without Ascites

Analysis of biochemical parameters did not show statistically significant differences between AC patients with and without ascites or between AC patients with different ascites degrees (Table 2). The analyzed parameters (bilirubin, AST, ALT, AP, GGT, and INR) were elevated in AC patients with and without ascites above the established normal values, except for creatinine and albumin, which remained within the average normal ranges. 

A statistically significant increase (*p* = 0.015) was observed when comparing alkaline phosphatase (AP) in AC patients with (176.41 ± 110.41 U/I) and without (172.80 ± 124.45 U/I) ascites. Regarding the INR (international normalized ratio), slightly elevated values were observed in AC patients with ascites (1.46 ± 0.34) compared to AC patients without ascites (1.38 ± 0.38). We also found a statistically significant increase in INR with increased ascites degree (*p* = 0.038). 

### 3.3. Analysis of KIR Genes in AC Patients with and without Ascites

The frequency of the presence (KIR+) or absence (KIR-) of KIR genes in control (N = 319) and AC patients (N = 281) was analyzed and compared. We also compared KIR frequencies between AC patients with (n = 162) and without (n = 119) ascites.

#### 3.3.1. Analysis of the Inhibitory KIR (iKIR) Gene Frequencies in AC Patients with and without Ascites

The frequency of iKIR genes in AC patients and controls was also analyzed (Table 3). No significant differences were detected in the univariate analysis of the frequency of iKIR genes between controls and patients, except for the KIR2DL2 gene, the frequency of which was significantly lower in total AC patients than in healthy controls (63.3% vs. 53.0%, respectively; OR = 0.654; 95% CI: 0.472–0.906, *p* = 0.013). In addition, the frequency of KIR2DL2 appears to be significantly decreased in AC patients with ascites compared with the control group (OR = 0.632; 95% CI: 0.431–0.927, *p* = 0.024). The frequency of other iKIR genes was similar between patients and controls. However, the KIR3DL1 gene slightly decreased in AC patients without ascites compared with those with ascites (93.2% vs. 98.3%, respectively; OR = 4.262; 95% CI: 0.927–19.599, *p* = 0.048).

#### 3.3.2. Analysis of the Frequencies of aKIR Genes in AC Patients with and without Ascites

Eleven aKIR genes were analyzed in AC patients and controls (Table 4). A similar frequency of aKIR genes was observed, except for KIR2SD2+ and KIR2SD5+ genes. The presence of KIR2SD2+ was significantly lower in AC patients than in controls (52% and 63%, respectively; OR = 0.635; 95% CI: 0.458–0.880, *p* = 0.006). In addition, when comparing AC patients without ascites (51.9%), a lower frequency was found compared to controls (63%); this difference was statistically significant (OR = 0.639; 95% CI: 0.417–0.977, *p* = 0.048). Similarly, the frequency of KIR2DS2+ genes among AC patients with ascites (51.9%) was significantly decreased compared to controls (63%, OR = 0.632; 95% CI: 0.431–0.927, *p* = 0.024).

The frequency of KIR2SD5 was significantly higher in AC patients than in controls (35.2% and 27%, respectively; OR = 1.474; 95% CI: 1.041–2.087, *p* = 0.033) and among AC patients without ascites (39.5%) (OR = 1.769; 95% CI: 1.136–2.754, *p* = 0.014); this difference was statistically significant.

### 3.4. Analysis of KIR Genes in AC Patients with Different Ascites Degrees

Next, the frequencies of KIR genes in AC patients with different degrees of ascites were analyzed. Among AC patients with ascites (N = 162), degrees I (n = 32), II (n = 66), and III (n = 56) were distinguished.

#### 3.4.1. Analysis of the Frequencies of iKIR Genes in AC Patients with Different Ascites Degrees 

As shown in Table 5, the frequency of iKIR genes was similar among AC patients with ascites of different degrees, with no significant differences detected in any of the iKIR genes. A significantly increased frequency of KIR3DL1+ was observed among AC patients with grade II ascites relative to grade I patients (95.5% compared to 87.5% respectively; *p* = 0.211). On the other hand, the control groups was compared with AC patients with different ascites grades. In most cases, the frequency was similar, although it was found that the frequencies of iKIR genes were decreased in AC patients with degree I ascites, with no significant results except for the KIR2DL2 gene. As shown in Table 4, the frequency of KIR2DL2 genes was decreased in AC patients with degree I ascites (43.8%) compared with the control group (63.3%); this difference was statistically significant (*p* = 0.036). The KIR2DL2 gene frequency in AC patients with degree II and III ascites was lower (51.5% and 51.8%, respectively).

#### 3.4.2. Analysis of the Frequencies of aKIR Genes in AC Patients with Different Degrees of Ascites

Similar results were obtained in the analysis of the aKIR genes, without significant differences between the frequencies among AC patients with ascites according to their grade (Table 6). Differences in the frequencies were obtained between patients with different degrees of ascites and the control group. The frequency of the KIR2DS2 gene was decreased in patients with grade I, II, and III ascites (43.8%, 51.5%, and 50%, respectively) compared to controls (63%). This difference was statistically significant only for the case of grade I ascites (*p* = 0.037). On the other hand, the frequency of the KIR2DS3 gene was increased in patients with grade I ascites (43.8%) compared with the control group (33.5%), while in individuals with grade II and III ascites, it was decreased (27.3% and 30.4%, respectively) in a non-significant manner. Finally, the KIR2DS5 gene was increased in patients with grade I (31.3%), II (34.8%), and III (30.4%) ascites compared with the control group (27%) but without statistical significance (Table 5).

### 3.5. Analysis of the Frequency of Epitopes and HLA-C Genotypes in AC Patients with Different Degrees of Ascites

Due to the importance of these Asn and Lys epitopes in the interaction of the HLA-C genotype as a ligand for KIR2D receptors, the possible influence of the presence of these epitopes in the population of AC patients and controls was studied (Table 7).

We observed that the number of patients lacking the C1 epitope was significantly decreased in AC patients compared to controls (OR = 0.586; 95% CI: 0.387–0.889, *p* = 0.015). Similar results were observed when analyzing AC patients with and without ascites compared to controls (OR = 0.548; 95% CI: 0.324–0.927, *p* = 0.031). However, analysis of the presence or absence of the C2 epitope did not show differences between controls and AC patients.

A subsequent analysis of HLA-C genotypes was performed on the same cohort of patients, demonstrating a similar frequency of the C1C1 genotype in controls and AC patients. The C1C2 and C2C2 genotypes were differently in the two groups(Table 7).

Likewise, the heterozygous C1C2 genotype was decreased in the population of patients without ascites compared with the control group (38.9% vs. 52.5%), presenting statistically significant differences (OR = 0.576; 95% CI: 0.372–0.893, *p* = 0.016). In addition, significant differences were obtained when comparing individuals with and without associated ascites, with an increase in the heterozygous C1C2 genotype in patients with associated ascites (OR = 1.670; 95% CI: 1.023–2.725, *p* = 0.048). However, this difference did not reach statistical significance despite showing a lower frequency in our patient population than in controls (46.3% and 52.5%, respectively).

On the other hand, the homozygous C2C2 genotype appeared to significantly increase in AC patients compared with the control group (23.5% vs. 15.3%, *p* = 0.015). Similar results were obtained when comparing the control group with AC patients without ascites (24.8% and *p* = 0.031). A lower frequency was also obtained in AC patients with ascites (22.6%) compared with the control group, at a borderline level of significance (*p* = 0.056). Subsequently, a frequency analysis of HLA-C epitopes and genotypes was performed in AC patients according to the degree of ascites. A similar frequency was observed among the groups (Table 8).

### 3.6. Analysis of KIR Gene Combinations and Their Corresponding HLA-C Ligands

Subsequently, the frequency of combinations of KIR genes with their known ligand was analyzed, considering the corresponding HLA-C ligand (C1, C2, or Bw4) in each case. For this analysis, inhibitory KIR genes and activating KIR genes were divided into two groups, and constitutive KIR genes and pseudogenes were excluded.

#### Analysis of KIR Genotypes and Their HLA-C Ligands in AC Patients with Ascites

As seen in Table 9, the iKIR genotype and its HLA-C ligands were evaluated in AC patients with ascites. The combination of KIR2DL1+/S1− in the presence and absence of its C2 epitope showed that the frequency of these combinations was similar in healthy controls and AC patients. On the contrary, when KIR2DL2 and KIR2DL3 associated with the C1+ ligand were analyzed, a lower frequency of these combinations was observed in the group of AC patients (83.5% vs. 76.2% and 85.1% vs. 75.9%, respectively), although this decrease was only significant for the combination of KIR2DL3 with C1+ (OR = 0.550; 95% CI: 0.353–0.858, *p* = 0.010). Similar results were obtained in the group of AC patients without ascites, but they were not significant.

Similarly, the frequency of the KIR3DL1+ gene in the presence and absence of its ligand, the BW4+ epitope, was also analyzed, but in this case, the frequencies were similar between the control group and AC patients (*p* = 0.846).

Regarding aKIR, the frequency of KIR2DS1+ in the presence and absence of the C2 epitope was also analyzed, finding no statistically significant differences between the control group and the patient population. The analysis of the combination of the KIR2DS4 gene with its two known ligands (C1+ and C2+) showed that the number of patients carrying the C1+ ligand and KIR2DS4 decreased compared to the control group (84.8% vs. 76.3%), and this difference was significant (OR = 0.575 95% CI: 0.376–0.880, *p* = 0.013). Something similar occurred when comparing AC patients without ascites with controls (75.2% vs. 84.8%), but the results were not statistically significant. In addition, the combination of KIR2DS5 with the C1+ ligand was less frequent in the whole group of patients with cirrhosis compared with the control group (72.9% vs. 84.3%), without statistical significance. In contrast, the combination of the C2+ ligand with KIR2DS5 appeared to increase in the patient group (74% vs. 67.5%), but the difference observed here was not statistically significant either. The results obtained in the group of AC patients with different ascites grades were insignificant.

## 4. Discussion

This study aimed to analyze the KIR/HLA-C genetic profile in AC patients with and without ascites to clarify the understanding of this pathology and find predictive clinical susceptibility biomarkers that can help to establish risks and prevent ascites development in AC patients. We performed a genetic study including controls and AC patients with and without ascites and analyzed the frequency of KIR and HLA-C genes and their combinations according to ligand–receptor compatibility to determine if there is any relationship with the risk of developing ascites or, on the contrary, a protection factor.

Evidence suggests that NK cells, via their membrane receptors, play dual roles in the development and progression of liver fibrosis, including profibrotic and antifibrotic functions [33], by selectively killing early or senescence-activated hepatic stellate cells (HSCs) and producing antifibrotic cytokine IFN-γ [34,35].

On the other hand, chronic ethanol use reduces the antifibrotic actions of NK cells/IFN-/STAT1 in the liver, suggesting novel and alternative therapeutic targets for the treatment of alcoholic liver fibrosis [36]. However, it is unknown whether the receptor–ligand couple (KIR/HLA) interacts directly in the alcohol metabolic pathway.

Our data show that AC patients had a decreased frequency of the iKIR2DL2 gene. The aKIR gene seems to influence the development of AC in patients. On the other hand, the increase in the KIR2DS5 gene was significant among AC patients compared with the control group. In our cohort of patients without ascites, the presence of KIR2SD5 was also significantly higher compared with the control group. Another study [22] found that KIR2DL2 and KIR2DS5 had the opposite impact in AC patients older than 54 years, with KIR2DL2 being protective against cirrhosis. In contrast, the presence of KIR2DS5 appears to encourage the fibrotic process, particularly in individuals without concomitant viral infection.

However, in other affectations, such as hepatoxicity, the combinations of KIR receptor–HLA ligand do not seem to have an effect [37]. KIR2DL2 and KIR3DL1 suggest a predisposition to the development of ascites. This suggests that a decrease in the KIR2DL2 gene, in addition to influencing AC development, could predispose patients to the development of ascites, since, on the one hand, the comparison between AC patients and controls was significant and, on the other hand, the comparison of AC patients without ascites and controls showed a lower frequency of KIR2DL2 but without significant results.

However, the patients without associated ascites presented a significantly lower KIR2DS2 frequency than controls, with a higher KIR2DS5 gene frequency in AC patients without ascites than controls. The same occurred with the KIR2DS2 gene. Patients with grade II and III ascites had a lower presence of both genes but without statistical significance. Thus, when KIR2DS2 was absent, there was a 100% chance that KIR2DL2 was also absent and an 82.39% chance that KIR2DS4 was present [38]. Other studies show a significantly increased frequency of KIR2DS5 in cirrhotic patients without associated viral infection compared to controls, with a similar trend in KIR3DS1+. On the other hand, a decrease in the frequency of KIR2DS5 in patients with viral infection compared to patients without viral infection was reported, suggesting a role in the development of a viral infection. However, in a joint analysis of the three centromeric KIR genes, they seemed to be more frequently represented in the population of AC patients and were suggested to participate in the biological mechanisms of the cirrhotic process [39].

The presence of KIR3DS1 receptors and KIR2DS1 has also been related to an increased risk of suffering from certain autoimmune diseases, such as ankylosing spondylitis [40], or, in the presence of the KIR2DL5 gene, as predisposing factors in multiple sclerosis [41]. Our data are similar to those published by Legaz et al. [22], which point to the KIR2DL1+ and KIR3DL2+ genes as factors of susceptibility to the development of ascites in AC patients. Similar data were obtained in both groups for KIR2DL1. However, other studies have found a relationship between the increased frequency of the KIR2DL2 gene and predisposition to the development of autoimmune diseases [40,41] or infectious diseases [42,43]. Other authors reported that ascites did not influence AC [44]. Due to the importance of these Asn and Lys epitopes in the interaction of the HLA-C genotype as a ligand for KIR receptors, the possible influence of the presence of these epitopes in AC patients and controls was studied, as well as their influence on the presence of AC and ascites.

Thus, it was observed that the number of patients lacking the C1 epitope was significantly decreased in the AC patients compared with the control group. All this suggests that the C1+ epitope did not influence the development of ascites but influenced the cirrhotic process. However, other studies have associated the HLA-C1 ligand group with an increased risk of systemic lupus erythematosus [45], inflammatory diseases [46], and HIV infection [47,48]. On the other hand, the heterozygous C1C2 genotype presented significant differences, with a lower frequency in patients without ascites compared with the control group. Significant differences were also obtained when comparing AC patients with and without ascites, with increased presence of the heterozygous C1C2 genotype in AC patients with ascites.

On the other hand, the frequency of the homozygous C2C2 genotype appeared to be significantly increased in AC patients compared with the control group. Similar results were obtained when comparing controls with AC patients with and without ascites. No differences were found regarding the degree of ascites.

Thiruchelvam-Kyle et al. [49] discovered a novel, as-yet-unidentified ligand for the KIR2DS2 NK cell activation receptor. Target cell HLA-C typing revealed that KIR2DS2 recognition was independent of HLA C1 and C2 groups, whereas targeting cells revealed that KIR2DL3 exclusively recognized expressed C1 group alleles. This coincides with our data, with reference to the population of AC patients that presents a reduction in the C1 epitope, as well as a lower frequency of the KIR2DL3 gene.

In another study, the combination these two factors was pointed out as a risk factor for human papillomavirus infection and the development of cervical cancer [50]. In contrast, the KIR2DL3/C1C2 genotype correlates with nodular melanoma and ulceration. In addition, the KIR2DL1(+)/S1(-)/C2C2 genotype, which is associated with susceptibility to melanoma and lymph node metastasis [51,52], was also observed with a highly significant increase in homozygous KIR2DL3/HLA-C1 in patients with Crohn’s disease, confirming the relevance of the KIR2DL2/KIR2DL3 genes and their interaction with HLA-C and disease. Another study suggested that KIR2DL2/KIR2DL3 heterozygosity in individuals carrying HLA-C2 increases the risk of certain diseases that involve inflammation, such as Chlamydia trachomatis infection [46]. Heterozygosity in individuals carrying HLA-C2 was also indicated as a risk factor for psoriasis associated with the HLA-C1 ligand versus KIR2DL2 in homozygosis [53]. KIR2DS2 and its HLA-C1 ligand have also been correlated with the pathogenesis of Hashimoto’s thyroiditis [54]. On the contrary, the predominance of the inhibitory interaction between KIR2DL2/3 receptors and HLA-C1 ligands in the absence of KIR2DS2 suggests a possible protective role in the pathogenesis of this disease. In contrast, for KIR2DS4+, Umemura et al. [55] presented significant associations with the HLA-C2 ligand, showing a significant progression toward liver cirrhosis. Ursu et al. [56] suggest that the expression of KIR2DL3, KIR2DL5, and KIR2DS4 and the association with HLA alleles may increase the patient’s susceptibility to developing chronic HCV infection. HLA-A*23:01 was the most frequent, although HLA-B* 44:02 and C*04:02 were also significantly elevated in HCV-positive patients. Kermes [57] suggests that the combination of KIR2DL3 + HLA-C1 genes with little inhibitory effect resolves infection with hepatitis C virus more efficiently.

A significant relationship between the KIR genes and their HLA ligands has also been observed with the incidence rate of SCD in the Iranian population [58]. Likewise, the frequency of the KIR3DL1+ gene in the presence and absence of its ligand, the Bw4+ epitope, was also analyzed, but in this case, the frequencies were similar between controls and AC patients. Another source describes the KIR3DL1 + HLA-Bw4 association as protective in patients with multiple sclerosis [59], as do García-León et al. [41], confirming HLA-Bw4 transport as a protective factor in multiple sclerosis. We observed significant results when analyzing the frequency of KIR2DS1+ in the presence and absence of the C2+ epitope. On the other hand, it has been pointed out that the stronger inhibitory interaction of KIR2DL1 + HLA-C2 affects placental development, causing reproductive problems such as pre-eclampsia, recurrent spontaneous abortion, and fetal growth restriction [60].

Finally, it should be noted that our study is subject to a series of limitations, among which is the lack of knowledge about the type of alcohol consumed, intensity, quantity, or even the age of onset of consumption. The sample size used in this genetic association study is appropriate both for the group of AC patients and the control group. Although a larger cohort of AC patients with ascites would have been desirable, it was possible to carry out all the statistical treatments in this study with no limitation in the results due to the number of patients analyzed. However, it was difficult to analyze the patient population according to the degree of ascites due to the small sample size. On the other hand, the results of this study can only be applied to other populations with control with a similar KIR/HLA gene repertoire due to the large differences in KIR/HLA gene repertoires between populations around the world. 

Furthermore, we only studied the KIR and HLA genes. Beyond the KIR and HLA genes, analysis of SNPs in genes for alcohol metabolism and immune function (e.g., PNPLA3, TM6SF2) [61,62,63,64,65], expression of liver fibrosis microRNAs (miR-122, miR-34a) [66,67,68], and epigenetic methylation patterns and genetic polymorphisms affecting oxidative stress [69,70] could provide further insight into alcoholic cirrhosis and ascites. In addition, examination of inflammation-regulating cytokine gene variants, a broader range of HLA alleles, and expanded KIR haplotypes, with integration of multiomic data on proteomic, metabolomic, and transcriptomic biomarkers, could significantly improve comprehensive identification of relevant genetic factors that influence pathogenesis. 

Given the obtained results, we emphasize the opportunities to find a more extensive profile of biomarkers, taking into account the age of first alcohol intake, alcohol consumption in grams per week, and post-transplant alcoholic recurrence in order to understand the etiology of AC and improve patient survival, on the one hand, and designing new tactics for the prevention and control of the abusive consumption of alcohol to detect, manage, and reduce the frequency of AC in our population through a deeper understanding of cirrhosis and ascites in future studies.

## 5. Conclusions

In conclusion, KIR2DS2, KIR2DS5, and KIR2DL2 genes are considered susceptibility factors for AC. A decrease in the KIR2DL2 and KIR3DL1 genes may be a predisposing factor for the development of ascites. The KIR2DS2 and KIR2DL2 genes may be involved in development of grade I ascites in AC patients. Regarding the association of KIR receptors and their ligands, the C1+ epitope does not seem to influence the development of ascites. The homozygous C2C2 genotype is a protective factor for AC patients without ascites. The C1C2 genotype may be a risk factor for ascites development in AC patients. The decrease in the combination of the KIR2DL3 genes with the C1+ ligand may influence the development of AC, while the combination of KIR2DS4 with the C1+ ligand may be considered a protective factor against AC.

## Figures and Tables

**Table 1:** Characterization of the study population of male AC patients.

		n (%)	Mean Age (Years ± SEM)
Total patients		281 (92.7)	53.63 ± 0.495
Ascites *	+	162 (57.7)	53.91 ± 0.644
	−	119 (42.3)	53.24 ± 0.775
Grade of Ascites	I	32 (20.7)	53.22 ± 1.381
	II	66 (43.2) ^a^	52.73 ± 1.001
	III	56 (36.1)	56.21 ± 1.074

N, total number of individuals; n, number of individuals in each subgroup; * Ascites, presence (+) or absence (−); SEM, standard error of the mean. Comparisons were made using a two-tailed Fisher’s exact test. The *p* value was obtained by comparing the group with grade II ascites with the rest of the groups. ^a^ The group with grade II ascites was compared with the rest of the groups (grade I and III ascites); OR = 0.349; 95% CI: 0.212–0.574, *p* < 0.001.

**Table 2:** Biochemical characteristics of male AC patients with and without ascites and grade of ascites.

		Male AC Patients				Grade of Ascites			
Parameter	Normal Values	Total Patients N = 281	Ascites + N = 162	Ascites − N = 119	P1	Grade I N = 91	Grade II N = 190	Grade III N = 190	P2
**Creatinine (mg/dL)**	0.7–1.2	228 *	155	73		30	64	55	
		1.09 ± 0.75 **	1.14 ± 0.81	0.99 ± 0.61	0.161	1.01 ± 0.26	1.11 ± 1.05	1.25 ± 0.74	0.413
**Albumin (g/dL)**	3.5–5.2	174	118	56		21	53	39	
		3.46 ± 0.66	3.34 ± 0.62	3.69 ± 0.68	0.139	3.46 ± 0.65	3.28 ± 0.60	3.40 ± 0.63	0.455
**Bilirubin total (mg/dL)**	0.05–1.2	180	124	56		25	52	41	
		3.15 ± 4.15	3.33 ± 4.00	2.74 ± 4.47	0.378	2.62 ± 1.78	2.87 ± 2.39	4.42 ± 6.14	0.3785
**AST (U/L)**	5–40	172	119	53		20	52	41	
		97.44 ±189.61	88.15 ± 101.21	118.30 ± 307.14	0.337	62.10 ± 47.54	102.36 ± 128.92	84.34 ± 83.10	0.114
**ALT (U/L)**	5–41	174	120	54		21	52	41	
		74.35 ± 158.57	66.60 ± 100.49	91.55 ± 242.83	0.338	72.90 ± 168.28	69.50 ± 89.13	60.43 ± 73.56	0.877
**AP (U/L)**	40–130	163	112	51		20	49	37	
		175.28 ± 114.61	176.41 ± 110.41	162.80 ± 124.45	**0.015**	155.65 ± 62.95	178.12 ± 121.85	163.32 ± 88.39	0.655
**GGT (U/L)**	10–71	161	111	50		19	50	37	
		103.07 ± 91.75	99.90 ± 95.66	110.10 ± 82.88	0.5156	75.42 ± 58.33	108.06 ± 92.82	93.13 ± 104.53	0.405
**INR**	0.9–1.2	215	148	67		30	59	55	
		1.44 ± 0.35	1.46 ± 0.34	1.38 ± 0.38	0.125	1.38 ± 0.20	1.42 ± 0.22	1.55 ± 0.46	**0.038**

* n, ** mean ± SEM; N, total number of individuals; n, number of individuals in each subgroup; SEM, standard error of the mean; AST, aspartate transaminase; ALT, alanine aminotransferase; AP, alkaline phosphatase; GGT, gamma-glutamyl transferase; IN, total number of individuals; SD, standard deviation; INR, international normalized ratio. Comparisons were made using Student’s t-test. P1, *p* value when comparing patients with and without ascites; P2, *p* value when comparing patients with ascites according to their grade; NR, international normalized ratio.

**Table 3:** Analysis of the frequency of iKIR genes in male AC patients and controls.

			Male AC Patients						
		Controls N = 319	Total AC Patients N = 281		Ascites − N = 119	Ascites + N = 162			
KIR Gene *	P/A	n (%)	n (%)	P1	n (%)	n (%)	P2	P3	P4
iKIRs									
**2DL1 (S1-)**	+	197 (61.8)	160 (56.9)	0.244	64 (53.8)	96 (59.3)	0.155	0.622	0.394
	−	122 (38.2)	121 (43.1)		55 (46.2)	66 (40.7)			
**2DL2**	+	202 (63.3)	149 (53.0)	**0.013 ^a^**	64 (53.8)	85 (52.5)	0.079	**0.024 ^b^**	0.904
	−	117 (36.7)	132 (47)		55 (46.2)	77 (47.5)			
**2DL3**	+	279 (87.5)	249 (88.6)	0.707	106 (89.1)	143 (88.3)	0.743	0.883	1.000
	−	40 (12.5)	32 (11.4)		13 (10.9)	19 (11.7)			
**2DL5**	+	170 (53.3)	158 (56.2)	0.511	70 (58.8)	88 (54.3)	0.322	0.847	0.468
	−	149 (46.7)	123 (43.8)		49 (41.2)	74 (45.7)			
**3DL1**	+	304 (95.3)	268 (95.4)	1.000	117 (98.3)	151 (93.2)	0.174	0.394	**0.048 ^c^**
	−	15 (4.7)	13 (4.6)		2 (1.7)	11 (6.8)			

P, presence; A, absence; N, total number of individuals; n, number of individuals with the presence or absence of a KIR gene. A two-tailed Fisher’s exact test was used for comparisons. P1, *p* value obtained by comparing total AC patients with controls; P2 and P3, *p* value obtained by comparing AC patients with and without ascites to controls, respectively; P4, *p* value obtained by comparing AC patients without and with ascites. ^a^ OR = 0.654; 95% CI:0.472–0.906, *p* = 0.013. ^b^ OR = 0.639; 95% CI: 0.436–0.938, *p* = 0.024 ^c^ OR = 4.262; 95% CI: 0.927–19.599, *p* = 0.048. * Housekeeping genes and pseudogenes are not included.

**Table 4:** Analysis of the frequency of aKIR genes in AC patients with and without ascites.

			Male AC Patients						
		Controls N = 319	Total Patients N = 281		Ascites − N = 119	Ascites + N = 162			
KIR Gene *	P/A	n (%)	n (%)	P1	n (%)	n (%)	P2	P3	P4
aKIRs									
**2DS1 (L1+)**	+	119 (37.3)	119 (42.3)	0.211	55 (46.2)	64 (39.5)	0.100	0.691	0.274
	−	200 (62.7)	162 (57.7)		64 (53.8)	98 (60.5)			
**2DS2 (L2+)**	+	201 (63.0)	146 (52.0)	**0.006 ^a^**	62 (52.1)	84 (51.9)	**0.048 ^c^**	**0.024 ^e^**	1.000
	−	118 (37.0)	135 (48)		57 (47.9)	78 (48.1)			
**2DS3**	+	107 (33.5)	93 (33.1)	0.931	41 (34.5)	52 (32.1)	0.910	0.838	0.702
	−	212 (66.5)	188 (66.9)		78 (65.5)	110 (67.9)			
**2DS4**	+	305 (95.6)	266 (94.7)	0.704	115 (96.6)	151 (93.2)	0.790	0.281	0.285
	−	14 (4.4)	15 (5.3)		4 (3.4)	11 (6.8)			
**2DS5**	+	86 (27.0)	99 (35.2)	**0.033 ^b^**	47 (39.5)	52 (32.1)	**0.014 ^d^**	0.243	0.209
	−	233 (73.0)	182 (64.8)		72 (60.5)	110 (67.9)			
**3DS1**	+	129 (40.4)	132 (47.0)	0.117	60 (50.4)	72 (44.4)	0.066	0.434	0.335
	−	190 (59.6)	149 (53.0)		59 (49.6)	90 (55.6)			

P, presence; A, absence; N, total number of individuals; n, number of individuals with the presence or absence of a KIR gene. A two-tailed Fisher’s exact test was used for comparisons. P1, *p* value obtained by comparing total AC patients with controls; P2 and P3, *p* value obtained by comparing AC patients with and without ascites to controls, respectively; P4, *p* value obtained by comparing AC patients without and with ascites. ^a^ OR = 0.635; 95% CI: 0.458–0.880, *p* = 0.006. ^b^ OR = 1.474; 95% CI: 1.041–2.087, *p* = 0.033. ^c^ OR = 0.639; 95% CI: 0.417–0.977, *p* = 0.048. ^d^ OR = 1.769; 95% CI: 1.136–2.754, *p* = 0.014. ^e^ OR = 0.632; 95% CI: 0.431–0.927, *p* = 0.024. * Housekeeping genes and pseudogenes are not included.

**Table 5:** Analysis of the frequency of iKIR genes in AC patients with different ascites degrees compared to controls.

			Male AC Patients										
					Grade of Ascites								
		Controls N = 319	Total Patients N = 281		Grade I N = 32	Grade II N = 66	Grade III N = 56						
KIR Gene *	P/A	n (%)	n (%)	P1	n (%)	n (%)	n (%)	P2	P3	P4	P5	P6	P7
iKIRs													
**2DL1 (S1-)**	+	197 (61.8)	160 (56.9)	0.244	16 (50)	41 (62.1)	34 (60.7)	0.281	0.375	1.000	0.254	1.000	0.883
	−	122 (38.2)	121 (43.1)		16 (50)	25 (37.9)	22 (39.3)						
**2DL2**	+	202 (63.3)	149 (53.0)	**0.013 ^a^**	14 (43.8)	34 (51.5)	29 (51.8)	0.523	0.512	1.000	**0.036 ^b^**	0.095	0.104
	−	117 (36.7)	132 (47)		18 (56.3)	32 (48.5)	27 (48.2)						
**2DL3**	+	279 (87.5)	249 (88.6)	0.707	26 (81.3)	61 (92.4)	51 (91.1)	0.169	0.198	1.000	0.406	0.299	0.655
	−	40 (12.5)	32 (11.4)		6 (18.8)	5 (7.6)	5 (8.9)						
**2DL5**	+	170 (53.3)	158 (56.2)	0.511	18 (56.3)	36 (54.5)	30 (53.6)	1.000	0.828	1.000	0.853	0.893	1.000
	−	149 (46.7)	123 (43.8)		14 (43.8)	30 (45.5)	26 (46.4)						
**3DL1**	+	304 (95.3)	268 (95.4)	1.000	28 (87.5)	63 (95.5)	52 (92.9)	0.211	0.455	0.702	0.083	1.000	0.504
	−	15 (4.7)	13 (4.6)		4 (12.5)	3 (4.5)	4 (7.1)						

P, presence; A, absence; N, total number of individuals; n, number of individuals with the presence or absence of a KIR gene. A two-tailed Fisher’s exact test was used for comparisons. P1, *p* value obtained by comparing total AC patients with controls; P2, *p* value obtained by comparing AC patients with grade I and II ascites; P3, *p* value obtained by comparing AC patients with grade I and III ascites; P4, *p* value obtained by comparing AC patients with grade II and III ascites; P5, P6, and P7, *p* value obtained by comparing controls with grade I, grade II, and grade III ascites, respectively. ^a^ OR = 0.654; 95% CI: 0.472–0.906, *p* = 0.013. ^b^ OR = 0.450; 95% CI: 0.216–0.939, *p* = 0.036. * Housekeeping genes and pseudogenes are not included. Data obtained from [32].

**Table 6:** Frequency of aKIR genes in AC patients with different degrees of ascites compared to controls.

			Male AC Patients										
					Grade of Ascites								
		Controls N = 319	Total Patients N = 281		Grade I N = 32	Grade II N = 66	Grade III N = 56						
KIR Gene *	P/A	n (%)	n (%)	P1	n (%)	n (%)	n (%)	P2	P3	P4	P5	P6	P7
aKIRs													
**2DS1 (L1+)**	+	119 (37.3)	119 (42.3)	0.211	15 (46.9)	25 (37.9)	21 (37.5)	0.511	0.500	1.000	0.341	1.000	1.000
	−	200 (62.7)	162 (57.7)		17 (53.1)	41 (62.1)	35 (62.5)						
**2DS2 (L2+)**	+	201 (63.0)	146 (52.0)	**0.006 ^a^**	14 (43.8)	34 (51.5)	28 (50)	0.523	0.659	1.000	**0.037 ^c^**	0.096	0.075
	−	118 (37.0)	135 (48)		18 (56.3)	32 (48.5)	28 (50)						
**2DS3**	+	107 (33.5)	93 (33.1)	0.931	14 (43.8)	18 (27.3)	17 (30.4)	0.114	0.249	0.841	0.249	0.387	0.758
	−	212 (66.5)	188 (66.9)		18 (56.3)	48 (72.7)	39 (69.6)						
**2DS4**	+	305 (95.6)	266 (94.7)	0.704	28 (87.5)	63 (95.5)	52 (92.9)	0.211	0.455	0.702	0.070	1.000	0.325
	−	14 (4.4)	15 (5.3)		4 (12.5)	3 (4.5)	4 (7.1)						
**2DS5**	+	86 (27.0)	99 (35.2)	**0.033 ^b^**	10 (31.3)	23 (34.8)	17 (30.4)	0.821	1.000	0.700	0.678	0.230	0.627
	−	233 (73.0)	182 (64.8)		22 (68.8)	43 (65.2)	39 (69.6)						
**3DS1**	+	129 (40.4)	132 (47.0)	0.117	16 (50)	29 (43.9)	24 (42.9)	0.667	0.657	1.000	0.347	0.680	0.769
	−	190 (59.6)	149 (53.0)		16 (50)	37 (56.1)	32 (57.1)						

P, presence; A, absence; N, total number of individuals; n, number of individuals with the presence or absence of a KIR gene. A two-tailed Fisher’s exact test was used for comparisons. P1, *p* value obtained by comparing total AC patients with controls; P2, *p* value obtained by comparing AC patients with grade I and II ascites; P3, *p* value obtained by comparing AC patients with grade I and III ascites; P4, *p* value obtained by comparing AC patients with associated grade II and III ascites; P5, P6, and P7, *p* value was obtained by comparing controls with grade I, II, and III ascites. ^a^ OR = 0.635; 95% CI: 0.458–0.880, *p* = 0.006. ^b^ OR = 1.474; 95% CI: 1.041–2.087, *p* = 0.033. ^c^ OR = 2.190; 95% CI: 1.051–4.565, *p* = 0.037. * Housekeeping genes and pseudogenes were not included.

**Table 7:** Analysis of the frequencies of HLA-C epitopes and genotypes in male AC patients with and without ascites.

		Male AC Patients						
	Controls	Total Patients		Ascites −	Ascites +			
	N = 314	N = 272		N = 113	N = 159			
Epitope HLA-C	n (%)	n (%)	P1	n (%)	n (%)	P2	P3	P4
C1+	266 (84.7)	208 (76.5)	**0.015 ^a^**	85 (75.2)	123 (77.4)	**0.031 ^c^**	0.056	0.772
C1−	48 (15.3)	64 (23.5)		28 (24.8)	36 (22.6)			
C2+	213 (67.8)	190 (69.9)	0.655	72 (63.7)	118 (74.2)	0.485	0.168	0.081
C2−	101 (32.2)	82 (30.1)		41 (36.3)	41 (36.3)			
**Genotype** **HLA-C**								
C1C1	101 (32.2)	82 (30.1)	0.655	41 (36.3)	41 (25.8)	0.485	0.168	0.081
C1C2	165 (52.5)	126 (46.3)	0.137	44 (38.9)	82 (51.6)	**0.016 ^d^**	0.846	**0.048 ^f^**
C2C2	48 (15.3)	64 (23.5)	**0.015 ^b^**	28 (24.8)	36 (22.6)	**0.031 ^e^**	0.056	0.772

N, total number of individuals; n, number of individuals with the presence or absence of the HLA-C genotype. Comparisons were made using a two-tailed Fisher’s exact test. P1, *p* value obtained by comparing total AC patients with controls; P2 and P3, *p* value obtained by comparing AC patients without and with ascites to controls, respectively; P4, *p* value obtained by comparing AC patients without and with ascites. ^a^ OR = 0.586; 95% CI: 0.387–0.889, *p* = 0.015. ^b^ OR = 1.705; 95% CI: 1.125–2.584, *p* = 0.015. ^c^ OR = 0.548; 95% CI: 0.324–0.927, *p* = 0.031. ^d^ OR = 0.576; 95% CI: 0.372–0.893, *p* = 0.016. ^e^ OR = 1.825; 95% CI: 1.079–3.090, *p* = 0.031. ^f^ OR = 1.670; 95% CI: 1.023–2.725, *p* = 0.048.

**Table 8:** Analysis of HLA-C epitopes and genotypes in AC patients according to ascites degree.

	Grade I N = 32	Grade II N = 65	Grade III N = 55			
Epitope HLA-C	n (%)	n (%)	n (%)	P1	P2	P3
C1+	23 (71.9)	50 (76.9)	45 (81.8)	0.622	0.295	0.653
C1−	9 (28.1)	15 (23.1)	10 (18.2)			
C2+	23 (71.9)	51 (78.5)	40 (72.7)	0.612	1.000	0.524
C2−	9 (28.1)	14 (21.5)	15 (27.3)			
**Genotype HLA-C**						
C1C1	9 (28.1)	14 (21.5)	15 (27.3)	0.612	1.000	1.000
C1C2	14 (43.8)	36 (55.4)	30 (54.5)	0.388	0.388	1.000
C2C2	9 (28.1)	15 (23.1)	10 (18.2)	0.622	0.622	0.653

N, total number of individuals; n, number of individuals with the presence or absence of the HLA-C genotype. Comparisons were made using a two-tailed Fisher’s exact test. P1, *p* value obtained by comparing AC patients with grade I and II ascites; P2, *p* value obtained by comparing AC patients with grade I and III ascites; P3, *p* value obtained by comparing AC patients with grade II and III ascites.

**Table 9:** Analysis of KIR genotypes and their HLA-C ligands in controls and AC patients with and without ascites.

		Controls N = 319	Patient Totals N = 281		Ascites − N = 119	Ascites + N = 213		Grade I N = 32	Grade II N = 66	Grade III N = 56			
KIR Genes	HLA-I Ligand	n (%)	n (%)	P1	n (%)	n (%)	P2	n (%)	n (%)	n (%)	P3	P4	P5
iKIRs													
KIR2DL1+/S1−	C2+	221 (67.6)	188 (70.1)	0.530	71 (63.4)	117 (75.0)	0.043	23 (76.7)	51 (78.5)	40 (72.7)	0.105	0.144	1.000
	C2−	1 (25.0)	2 (50.0)		0 (0)	2 (66.7)		2 (100.0)	0 (0)	0 (0)			
KIR2DL2+	C1+	167 (83.5)	109 (76.2)	0.099	46 (76.7)	63 (75.9)	1.000	9 (64.3)	27 (77.1)	22 (78.6)	0.710	0.402	0.516
	C1−	15 (12.9)	30 (23.3)		14 (26.4)	16 (21.1)		4 (22.2)	8 (25.8)	4 (14.8)			
KIR2DL3+	C1+	235 (85.1)	183 (75.9)	**0.010 ^a^**	75 (75.0)	108 (76.6)	0.879	18 (69.2)	46 (76.7)	41 (80.4)	1.000	0.317	0.497
	C1−	7 (17.5)	6 (19.4)		3 (23.1)	3 (16.7)		1 (16.7)	2 (33.3)	0 (0)			
KIR3DL1+	Bw4+	231 (76.0)	201 (75.0)	0.846	87 (74.4)	114 (75.5)	0.887	21 (75.0)	47 (73.4)	40 (76.9)	0.647	0.610	1.000
	Bw4–	7 (46.7)	7 (53.8)		0 (0)	7 (63.6)		2 (50.0)	3 (100.0)	2 (50.0)			
**aKIRs**													
KIR2DS1+	C2+	78 (67.2)	81 (70.4)	0.670	32 (61.5)	49 (77.8)	0.067	10 (66.7)	21 (84.0)	17 (81.0)	1.000	0.734	0.598
	C2−	64 (32.0)	48 (30.6)		21 (34.4)	27 (28.1)		4 (23.5)	10 (24.4)	11 (32.4)			
	C2+	206 (68.0)	178 (69.3)	0.784	70 (64.2)	108 (730)	0.136	19 (67.9)	49 (77.8)	37 (72.5)	1.000	1.000	0.437
	C2−	5 (38.5)	3 (20.0)		2 (50.0)	1 (9.1)		0 (0)	0 (0)	1 (25.0)			
KIR2DS4+													
	C1+	257 (84.8)	196 (76.3)	**0.013 ^b^**	82 (75.2)	114 (77.0)	0.768	20 (71.4)	47 (74.6)	42 (82.4)	0.309	1.000	0.478
	C1−	2 (15.4)	3 (20.0)		1 (25.0)	2 (18.2)		1 (25.0)	0 (0)	1 (25.0)			
	C1+	70 (84.3)	70 (72.9)	0.072	31 (70.5)	39 (75.0)	0.651	8 (80.0)	17 (70.8)	12 (70.6)	0.517	0.467	1.000
	C1−	35 (15.0)	38 (21.6)		15 (21.7)	23 (21.5)		7 (31.8)	9 (21.4)	5 (13.2)			
KIR2DS5+													
	C2+	56 (67.5)	71 (74.0)	0.410	30 (68.2)	41 (78.8)	0.253	8 (80.0)	19 (79.2)	14 (82.4)	0.509	1.000	0.403
	C2−	75 (32.2)	57 (32.4)		27 (39.1)	30 (28.0)		7 (31.8)	9 (21.4)	12 (31.6)			

N, total number of individuals; n, number of individuals with the presence or absence of a ligand for a KIR gene. A two-tailed Fisher’s exact test was used for comparisons. P1, *p* value obtained by comparing total AC patients with controls; P2, *p* value obtained by comparing AC patients without and with ascites; P3, *p* value obtained by comparing AC patients with grade I and II ascites; P4, *p* value obtained by comparing AC patients with grade I and III ascites; P5, *p* value obtained by comparing AC patients with grade II and III ascites. ^a^ OR = 0.550; 95% CI: 0.353–0.858, *p* = 0.010; ^b^ OR = 0.575 95% CI: 0.376–0.880, *p* = 0.013.

## Data Availability

Not applicable.

## References

[1] Anstee Q.M., Seth D., Day C.P. (2016). Genetic Factors That Affect Risk of Alcoholic and Nonalcoholic Fatty Liver Disease. Gastroenterology.

[2] Arroyo V. (2002). Pathophysiology, diagnosis and treatment of ascites in cirrhosis. Ann. Hepatol..

[3] Arroyo V., Gines P., Gerbes A.L., Dudley F.J., Gentilini P., Laffi G., Reynolds T.B., Ring-Larsen H., Schölmerich J. (1996). Special Article Definition and Diagnostic Criteria of Refractory Ascites and Hepatorenal Syndrome in Cirrhosis. Hepatology.

[4] Bamias A., Tsiatas M., Kafantari E., Liakou C., Rodolakis A., Voulgaris Z., Vlahos G., Papageorgiou T., Tsitsilonis O., Bamia C. (2007). Significant differences of lymphocytes isolated from ascites of patients with ovarian cancer compared to blood and tumor lymphocytes. Association of CD3+CD56+ cells with platinum resistance. Gynecol. Oncol..

[5] Bamias A., Koutsoukou V., Terpos E., Tsiatas M.L., Liakos C., Tsitsilonis O., Rodolakis A., Voulgaris Z., Vlahos G., Papageorgiou T. (2008). Correlation of NK T-like CD3+CD56+ cells and CD4+CD25+(hi) regulatory T cells with VEGF and TNFα in ascites from advanced ovarian cancer: Association with platinum resistance and prognosis in patients receiving first-line, platinum-based chemotherapy. Gynecol. Oncol..

[6] Barquera R., Zuñiga J. (2008). Consortium for the Analysis of the Diversity and Evolution of Latinamerica View Project Immunogenetic Characterization of Central American Populations View Project. www.iner.gob.mx.

[7] Ursu L., Calenic B., Diculescu M., Dima A., Constantinescu I. (2020). HLA Alleles and KIR Genes in Romanian Patients with Chronic Hepatitis C. J. Gastrointestin. Liver Dis..

[8] Campillo J.A., Legaz I., López-Álvarez M.R., Bolarín J.M., Heras B.L., Muro M., Minguela A., Moya-Quiles M.R., Blanco-García R., Martínez-Banaclocha H. (2013). KIR gene variability in cutaneous malignant melanoma: Influence of KIR2D/HLA-C pairings on disease susceptibility and prognosis. Immunogenetics.

[9] Características Clínicas De Los Pacientes Con Cirrosis Internados En El Servicio De Clínica Médica|Revista Argentina De Medicina. (n.d.). http://www.revistasam.com.ar/index.php/RAM/article/view/261.

[10] Cárdenas A., Arroyo V. (2003). Mechanisms of water and sodium retention in cirrhosis and the pathogenesis of ascites. Best Pract. Res. Clin. Endocrinol. Metab..

[11] Cárdenas A., Bataller R., Arroyo V. (2000). Mechanisms of Ascites Formation. Clin. Liver Dis..

[12] Choudhary N.S., Duseja A. (2021). Genetic and epigenetic disease modifiers: Non-alcoholic fatty liver disease (NAFLD) and alcoholic liver disease (ALD). Transl. Gastroenterol. Hepatol..

[13] Chuang W.-L., Liu H.-W., Chang W.-Y., Chen S.-C., Hsieh M.-Y., Wang L.-Y. (1991). Natural killer cell activity in patients with liver cirrhosis relative to severity of liver damage. Dig. Dis. Sci..

[14] Colonna M., Borsellino G., Falco M., Ferrara G.B., Strominger J.L. (1993). HLA-C is the inhibitory ligand that determines dominant resistance to lysis by NK1- and NK2-specific natural killer cells. Proc. Natl. Acad. Sci. USA.

[15] Complications of Cirrhosis, an Issue of Clinics in Liver Disease, E-Book—Google Libros. (n.d.). https://books.google.es/books?hl=es&lr=&id=84cpEAAAQBAJ&oi=fnd&pg=PP1&dq=At+this+last+stage+of+alcoholic+cirrhosis,+patients+suffer+complications+associated+with+portal+hypertension,+including+ascites,+spontaneous+bacterial+peritonitis+(SBP),+hepatic+encephalopathy+(HE),+hepatorenal+syndrome,+portopulmonary+hypertension,+or+var&ots=_h1hq9PR35&sig=OeTLGYnvRXVFveOP1mnSLnbwSDs#v=onepage&q&f=false.

[16] Del Campo J.A., Gallego-Durán R., Gallego P., Grande L. (2018). Genetic and Epigenetic Regulation in Nonalcoholic Fatty Liver Disease (NAFLD). Int. J. Mol. Sci..

[17] Díaz-Peña R., Vidal-Castiñeira J.R., Moro-García M.A., Alonso-Arias R., Castro-Santos P. (2016). Significant association of the KIR2DL3/HLA-C1 genotype with susceptibility to Crohn’s disease. Hum. Immunol..

[18] Dumitrescu R.G. (2018). Alcohol-induced epigenetic changes in cancer. Methods Mol. Biol..

[19] EBSCOhost|87336999|Austrian Consensus on the Definition and Treatment of Portal Hypertension and Its Complications (Billroth II). (n.d.). https://eds.s.ebscohost.com/abstract?site=eds&scope=site&jrnl=00435325&AN=87336999&h=EkZoak1pq0P%2Bc%2B%2FW9MZp0Qm8MgzhuVVLxmJa8%2BhhJcWfTkKznvKSeeY6qBFTuRA0aYj3aRsH6Gu4vhBwbMC90w%3D%3D&crl=c&resultLocal=ErrCrlNoResults&resultNs=Ehost&crlhashurl=login.aspx%3Fdirect%3Dtrue%26profile%3Dehost%26scope%3Dsite%26authtype%3Dcrawler%26jrnl%3D00435325%26AN%3D87336999.

[20] Salerno F., Angeli P., Bernardi M., Laffi G., Riggio O., Salvagnini M. (1999). Clinical practice guidelines for the management of cirrhotic patients with ascites. Committee on Ascites of the Italian Association for the Study of the Liver. Ital. J. Gastroenterol. Hepatol..

[21] Franco S., Horneros J., Soldevila L., Ouchi D., Galván-Femenía I., de Cid R., Tenesa M., Bechini J., Perez R., Llibre J.M. (2021). Single nucleotide polymorphisms in PNPLA3, ADAR-1 and IFIH1 are associated with advanced liver fibrosis in patients co-infected with HIV-1//hepatitis C virus. AIDS.

[22] Gambino C.M., Di Bona D., Aiello A., Carru C., Duro G., Guggino G., Ferrante A., Zinellu A., Caruso C., Candore G. (2018). HLA-C1 ligands are associated with increased susceptibility to systemic lupus erythematosus. Hum. Immunol..

[23] Gao B., Radaeva S. (2013). Natural killer and natural killer T cells in liver fibrosis. Biochim. Biophys. Acta (BBA)-Mol. Basis Dis..

[24] García-León J.A., Pinto-Medel M.J., García-Trujillo L., López-Gómez C., Oliver-Martos B., Prat-Arrojo I., Marín-Bañasco C., Suardíaz-García M., Maldonado-Sanchez R., Fernández-Fernández Ó. (2011). Killer cell immunoglobulin-like receptor genes in Spanish multiple sclerosis patients. Mol. Immunol..

[25] Buey L.G., Mateos F.G., Moreno-Otero R. (2012). Cirrosis hepática. Med.-Programa Form. Médica Contin. Acreditado.

[26] Ginès P., Angeli P., Lenz K., Møller S., Moore K., Moreau R., Hayes P. (2010). EASL clinical practice guidelines on the management of ascites, spontaneous bacterial peritonitis, and hepatorenal syndrome in cirrhosis. J. Hepatol..

[27] Gourraud P.-A., Meenagh A., Cambon-Thomsen A., Middleton D. (2010). Linkage disequilibrium organization of the human KIR superlocus: Implications for KIR data analyses. Immunogenetics.

[28] Heidelbaugh J.J., Bruderly M. (2006). Cirrhosis and Chronic Liver Failure: Part I. Diagnosis and Evaluation. Am. Fam. Physician.

[29] Hiby S.E., Apps R., Sharkey A.M., Farrell L.E., Gardner L., Mulder A., Claas F.H., Walker J.J., Redman C.C., Morgan L. (2010). Maternal activating KIRs protect against human reproductive failure mediated by fetal HLA-C2. J. Clin. Investig..

[30] Hochreuter M.Y., Dall M., Treebak J.T., Barrès R. (2022). MicroRNAs in non-alcoholic fatty liver disease: Progress and perspectives. Mol. Metab..

[31] Hollenbach J.A., Pando M.J., Caillier S.J., Gourraud P.A., Oksenberg J.R. (2016). The killer immunoglobulin-like receptor KIR3DL1 in combination with HLA-Bw4 is protective against multiple sclerosis in African Americans. Genes Immun..

[32] Hou W., Sanyal A.J. (2009). Ascites: Diagnosis and Management. Med. Clin. N. Am..

[33] Hou Y.-F., Zhang Y.-C., Jiao Y.-L., Wang L.-C., Li J.-F., Pan Z.-L., Yang Q.-R., Sun H.-S., Zhao Y.-R. (2010). Disparate distribution of activating and inhibitory killer cell immunoglobulin-like receptor genes in patients with systemic lupus erythematosus. Lupus.

[34] Morales Penalva R. (2022). Ascitis y encefalopatía, causas de muerte y supervivencia del paciente por cirrosis alcohólica y su influencia inmunológica. Proy. Investig..

[35] Jeong W.I., Park O., Gao B. (2008). Abrogation of the Antifibrotic Effects of Natural Killer Cells/Interferon-γ Contributes to Alcohol Acceleration of Liver Fibrosis. Gastroenterology.

[36] Jiao Y.-L., Ma C.-Y., Wang L.-C., Cui B., Zhang J., You L., Chen Z.-J., Li J.-F., Zhao Y.-R. (2008). Polymorphisms of KIRs gene and HLA-C alleles in patients with ankylosing spondylitis: Possible association with susceptibility to the disease. J. Clin. Immunol..

[37] Kawaratani H., Fukui H., Yoshiji H. (2017). Treatment for cirrhotic ascites. Hepatol. Res..

[38] Khakoo S.I., Thio C.L., Martin M.P., Brooks C.R., Gao X., Astemborski J., Cheng J., Goedert J.J., Vlahov D., Hilgartner M. (2004). HLA and NK cell inhibitory receptor genes in resolving hepatitis C virus infection. Science.

[39] Legaz I., Bolarín J.M., Navarro E., Campillo J.A., Moya R., Pérez-Cárceles M.D., Luna A., Osuna E., Miras M., Muro M. (2021). KIR2DL2/S2 and KIR2DS5 in alcoholic cirrhotic patients undergoing liver transplantation. Arch. Med. Sci..

[40] Legaz I., López-Álvarez M.R., Campillo J.A., Moya-Quiles M.R., Bolarín J.M., de la Peña J., Salgado G., Gimeno L., García-Alonso A.M., Muro M. (2013). KIR gene mismatching and KIR/C ligands in liver transplantation: Consequences for short-term liver allograft injury. Transplantation.

[41] Legaz I., Navarro-Noguera E., Bolarín J.M., García-Alonso A.M., Maldonado A.L., Mrowiec A., Campillo J.A., Gimeno L., Moya-Quiles R., Álvarez-López M.d.R. (2016). Epidemiology, Evolution, and Long-Term Survival of Alcoholic Cirrhosis Patients Submitted to Liver Transplantation in Southeastern Spain. Alcohol. Clin. Exp. Res..

[42] Legaz I., Noguera E.N., Bolarín J.M., Campillo J.A., Moya R., Luna A., Miras M., Minguela A., Álvarez-López M.R., Muro M. (2018). Patient Sex in the Setting of Liver Transplant in Alcoholic Liver Disease. Exp. Clin. Transplant..

[43] Li J.-T., Guo C., Li M.-L., Wei Y.-Q., Hou Y.-F., Jiao Y.-L., Zhao Y.-R., Sun H., Xu J., Cao M.-F. (2016). Killer Cell Immunoglobulin-Like Receptor Genes and their HLA-C Ligands in Hashimoto Thyroiditis in a Chinese Population. Endocr. Pract..

[44] Lutz P., Jeffery H.C., Jones N., Birtwistle J., Kramer B., Nattermann J., Spengler U., Strassburg C.P., Adams D.H., Oo Y.H. (2019). NK cells in ascites from liver disease patients display a particular phenotype and take part in antibacterial immune response. Front. Immunol..

[45] Maclaren R. (2009). Management of Cirrhosis and Associated Complications Learning Objectives. J. Pharm. Pract..

[46] Mahmoudi M., Fallahian F., Sobhani S., Ghoroghi S., Jamshidi A., Poursani S., Dolati M., Hosseinpour Z., Gharibdoost F. (2017). Analysis of killer cell immunoglobulin-like receptors (KIRs) and their HLA ligand genes polymorphisms in Iranian patients with systemic sclerosis. Clin. Rheumatol..

[47] Moretta A., Vitale M., Bottino C., Orengo A.M., Morelli L., Augugliaro R., Barbaresi M., Ciccone E., Moretta L. (1993). P58 molecules as putative receptors for major histocompatibility complex (MHC) class I molecules in human natural killer (NK) cells. Anti-p58 antibodies reconstitute lysis of MHC class I-protected cells in NK clones displaying different specificities. J. Exp. Med..

[48] Mu T., Peng L., Xie X., He H., Shao Q., Wang X., Zhang Y. (2022). Single Nucleotide Polymorphism of Genes Associated with Metabolic Fatty Liver Disease. J. Oncol..

[49] Murray K.F., Carithers R.L. (2005). AASLD practice guidelines: Evaluation of the patient for liver transplantation. Hepatology.

[50] Nham T., Poznanski S.M., Fan I.Y., Shenouda M.M., Chew M.V., Lee A.J., Vahedi F., Karimi Y., Butcher M., Lee D.A. (2018). Ex vivo-expanded NK cells from blood and ascites of ovarian cancer patients are cytotoxic against autologous primary ovarian cancer cells. Cancer Immunol. Immunother..

[51] Parolini F., Biswas P., Serena M., Sironi F., Muraro V., Guizzardi E., Cazzoletti L., Scupoli M.T., Gibellini D., Ugolotti E. (2018). Stability and Expression Levels of HLA-C on the Cell Membrane Modulate HIV-1 Infectivity. J. Virol..

[52] Rajagopalan S., Long E.O. (2005). Understanding how combinations of HLA and KIR genes influence disease. J. Exp. Med..

[53] Rizzo R., Gentili V., Casetta I., Caselli E., De Gennaro R., Granieri E., Cassai E., Di Luca D., Rotola A. (2012). Altered natural killer cells’ response to herpes virus infection in multiple sclerosis involves KIR2DL2 expression. J. Neuroimmunol..

[54] Rizzo R., Gentili V., Rotola A., Bortolotti D., Cassai E., Di Luca D. (2014). Implication of HLA-C and KIR Alleles in Human Papillomavirus Infection and Associated Cervical Lesions. Viral Immunol..

[55] Roberts C.H., Molina S., Makalo P., Joof H., Harding-Esch E.M., Burr S.E., Mabey D.C.W., Bailey R.L., Burton M.J., Holland M.J. (2014). Conjunctival Scarring in Trachoma Is Associated with the HLA-C Ligand of KIR and Is Exacerbated by Heterozygosity at KIR2DL2/KIR2DL3. PLoS Neglected Trop. Dis..

[56] Salerno F., Guevara M., Bernardi M., Moreau R., Wong F., Angeli P., Garcia-Tsao G., Lee S.S. (2010). Refractory ascites: Pathogenesis, definition and therapy of a severe complication in patients with cirrhosis. Liver Int..

[57] Salvoza N.C., Klinzing D.C., Gopez-Cervantes J., Baclig M.O. (2016). Association of Circulating Serum miR-34a and miR-122 with Dyslipidemia among Patients with Non-Alcoholic Fatty Liver Disease. PLoS ONE.

[58] Sayaf K., Gabbia D., Russo F.P., De Martin S. (2022). The Role of Sex in Acute and Chronic Liver Damage. Int. J. Mol. Sci..

[59] Serena M., Parolini F., Biswas P., Sironi F., Miranda A.B., Zoratti E., Scupoli M.T., Ziglio S., Valenzuela-Fernandez A., Gibellini D. (2017). HIV-1 Env associates with HLA-C free-chains at the cell membrane modulating viral infectivity. Sci. Rep..

[60] Shi J., Zhao J., Zhang X., Cheng Y., Hu J., Li Y., Zhao X., Shang Q., Sun Y., Tu B. (2017). Activated hepatic stellate cells impair NK cell anti-fibrosis capacity through a TGF-β-dependent emperipolesis in HBV cirrhotic patients. Sci. Rep..

[61] Stephens C., Moreno-Casares A., López-Nevot M., García-Cortés M., Medina-Cáliz I., Hallal H., Soriano G., Roman E., Ruiz-Cabello F., Romero-Gomez M. (2016). Killer immunoglobulin-like receptor profiles are not associated with risk of amoxicillin-clavulanate-induced liver injury in Spanish patients. Front. Pharmacol..

[62] Stickel F., Moreno C., Hampe J., Morgan M.Y. (2017). The genetics of alcohol dependence and alcohol-related liver disease. J. Hepatol..

[63] Thiruchelvam-Kyle L., Hoelsbrekken S.E., Saether P.C., Bjørnsen E.G., Pende D., Fossum S., Daws M.R., Dissen E. (2017). The Activating Human NK Cell Receptor KIR2DS2 Recognizes a β2-Microglobulin–Independent Ligand on Cancer Cells. J. Immunol..

[64] Tonetti C.R., de Souza-Araújo C.N., Yoshida A., da Silva R.F., Alves P.C.M., Mazzola T.N., Derchain S., Fernandes L.G.R., Guimarães F. (2021). Ovarian cancer-associated ascites have high proportions of cytokine-responsive CD56bright NK cells. Cells.

[65] Umemura T., Joshita S., Saito H., Wakabayashi S.-I., Kobayashi H., Yamashita Y., Sugiura A., Yamazaki T., Ota M. (2021). Investigation of the Effect of KIR–HLA Pairs on Hepatocellular Carcinoma in Hepatitis C Virus Cirrhotic Patients. Cancers.

[66] Van den Besselaar A.M.H.P. (1996). Precision and accuracy of the international normalized ratio in oral anticoagulant control. Haemostasis.

[67] Varela-Rey M., Woodhoo A., Martinez-Chantar M.L., Mato J.M., Lu S.C. (2013). Alcohol, DNA Methylation, and Cancer. Alcohol Res. Curr. Rev..

[68] WHO (2018). Global Status Report on Alcohol and Health 2018.

[69] Yamada H., Suzuki K., Ichino N., Ando Y., Sawada A., Osakabe K., Sugimoto K., Ohashi K., Teradaira R., Inoue T. (2013). Associations between circulating microRNAs (miR-21, miR-34a, miR-122 and miR-451) and non-alcoholic fatty liver. Clin. Chim. Acta.

[70] Yang M., Vanderwert E., Kimchi E.T., Staveley-O’Carroll K.F., Li G. (2023). The Important Roles of Natural Killer Cells in Liver Fibrosis. Biomedicines.

